# Availability of Adequately Iodized Dietary Salt and Associated Factors in a Town of Southeast Ethiopia: A Community-Based Cross-Sectional Survey

**DOI:** 10.1155/2020/1357192

**Published:** 2020-12-04

**Authors:** Gadisa Fitala Obssie, Kassahun Ketema, Yohannes Tekalegn

**Affiliations:** ^1^Gobe Health Center, West Arsi Health Department, Oromia Regional Health Bureau, Kore Town, Ethiopia; ^2^Department of Public Health, School of Health Science, Goba Referral Hospital, Madda Walabu University, Bale Robe, Ethiopia

## Abstract

**Background:**

Iodine deficiency is the world's major cause of preventable intellectual impairment, and nearly 2 billion people are at risk of iodine deficiency worldwide. Prevention and control of iodine deficiency disorders primarily aim at ensuring the intake of adequate iodine to maintain normal thyroid function. In our study area, studies regarding the coverage of adequately iodized salt at household level are meager. Hence, this study aimed to assess the magnitude of adequately iodized dietary salt at a household level in Kore Town, Southeast Ethiopia.

**Methods:**

A community-based cross-sectional study was conducted in the Kore town from August 1 to 30, 2019. A total of 394 households were selected for this study using a simple random sampling technique. The level of salt iodine content was determined using the rapid field test kit. Then, iodine contents of dietary salt were reported as <15 parts per million and ≥15 parts per million. Data regarding sociodemographic factors, knowledge of respondents about iodized salt, and iodized salt handling practices were collected through a face-to-face interview. The binary logistic regression model was used to assess the association between independent factors and the outcome variable. Statistical significance was declared at *p* < 0.05.

**Result:**

Out of all the households, 223 (56.6%) had adequately iodized salt. Not exposing iodized salt to sunlight (AOR = 2.35, 95% CI: 1.1, 5.2), storing the salt at a dry or cold place [(AOR = 4.77, 95% CI: 1.39, 16.45) and (AOR = 8.23, 95% CI: 1.44, 47.19), respectively], and having good knowledge about iodized salt (AOR = 1.88, 95% CI: 1.18, 3.01) were significantly associated with the presence of adequately iodized salt at the household level.

**Conclusion:**

Availability of adequately iodized salt in the study area was far below the World Health Organization recommendation. Information regarding the importance and proper handling of iodized dietary salt should be communicated to the householders.

## 1. Background

Iodine is an essential element needed for life. It plays a vital role in the production of thyroid hormone in humans as well as in all vertebrates. Iodine deficiency can lead to serious health problems, including goiter, mental retardation, and cretinisms [1–4]. Iodine deficiency is a public health problem throughout the world. Globally, about 2 billion people are at risk of iodine deficiency [5, 6]. In developing countries, 38 million newborn babies per year are at risk of the devastating consequences of iodine deficiency [7]. In Ethiopia, 62% of the people are at risk of iodine deficiency [8].

The World Health Organization recommends a daily iodine intake of 50 micrograms for infants, 90 micrograms for preschool children, 120 micrograms for school children, 150 micrograms for adults, and 250 micrograms for pregnant and lactating mothers to prevent iodine deficiency disorders [9, 10]. Despite this, one-third of the world population lives in the area with some iodine deficiency [11].

Prevention and control of iodine deficiency disorders primarily aim at ensuring adequate intake of dietary iodine. Increased dietary iodine intake can be implemented through food fortification with iodine. Salt is the most commonly used vehicle since it is inexpensive and widely available. The World Health Organization recommends eliminating iodine deficiency disorders through universal salt iodization [1].

Coverage of iodized salt shows a gradual improvement in Ethiopia as it was 28.4% in 2000 [12], 54.3% in 2005 [13], 15.4% in 2011 [14], 88.8% in 2014 [15], and 89% in 2016 [16]. However, disparities exist in terms of adequacy of the iodine level in dietary salt and its utilization level within and between different regions of the country [15]. In Ethiopia, household surveys of salt iodine levels were conducted at a different time and reported a low level of adequately iodized salt [15, 17–21].

To eliminate iodine deficiency disorders, the World Health Organization recommends that at least 90% of households should be able to access adequately iodized salt [1]. Assessing the proportion of households with access to adequately iodized dietary salt is very essential in tracking progress towards universal salt iodization. In this aspect, there are no studies conducted in our study area that assess the level of adequately iodized salt at household level. Therefore, this study aimed to assess the magnitude and factors associated with availability of adequately iodized dietary salt at household level in Kore Town, Southeast Ethiopia.

## 2. Materials and Methods

### 2.1. Study Design and Setup

A community-based cross-sectional study was conducted in the Kore town from August 01 to 30, 2019. The town is located at 49 kilometers in the northeastern direction of Shashemene town, capital of West Arsi zone, Southeast Ethiopia. The town has a total of 1033 households and about 4956 total population. The major crops produced in the woreda include wheat, barley, bean, pea, potato, inset, cabbage, onion, and maize.

### 2.2. Sample Size Determination and Sampling Procedure

The sample size of the study was calculated using Epi Info version 7 software. The following assumptions were used: the proportion of households with adequately iodized salt was 63% [22], 95% confidence level, and a 5% margin of error. The final sample size was 394 households, after adding a 10% contingency for possible nonresponse. The samples of households were selected randomly from the list of all households in the town using a simple random sampling technique. The sampling frames of the households were obtained from the town's health department.

### 2.3. Data Collection Tools, Procedure, and Quality Assurance

A structured, interviewer-administered questionnaire was used to collect the data. The questionnaire was first prepared in English and translated into the local language (Afan Oromo) and finally backtranslated to English to ensure consistency. The questionnaire contains queries regarding sociodemographic variables, knowledge of iodized salt, and utilization of iodized salt. The questionnaires were pretested on five percent of the sample before the actual data collection. Eight data collectors and two supervisors were recruited for the study. The data collectors were health professionals with a bachelor's degree, and the supervisors were public health professionals with a master's degree. Two days of training were given for data collectors and supervisors regarding data collection techniques. Completeness of questionnaires was checked daily throughout the data collection period.

### 2.4. Determination of the Iodine Content of the Salt

A tablespoon full of salt was collected from each sampled household, and the rapid test kit (RTK) was used to determine the level of salt iodine content. The small cup in the kit was filled with salt, and the cup surface was made flat. Two drops of the test solution from a white ampoule were added to the surface of the salt by piercing the white ampoule with a pin and gently squeezing the ampoule. The iodine content in the salt was determined within one minute by comparing the color change on the salt against the standard color chart. If no color appears after 1 minute, 5 drops of the recheck solution from the red ampoule was added to a fresh salt sample and followed by 2 drops of the test solution on the same salt sample. Then, a comparison was done with the standard color chart [1]. Based on the reading from the standard color chart values, the samples were categorized into <15 parts per million (inadequately iodized salt) or ≥15 parts per million (adequately iodized salt) [1].

### 2.5. Dependent Variable

#### 2.5.1. Availability of Adequately Iodized Salt in Household

As described above, availability of adequately iodized salt was dichotomized into two categories either as adequately iodized or inadequately iodized.

#### 2.5.2. Independent Variables

Independent variables include variables related with sociodemographic characteristics of householders, iodized salt handling practices, and knowledge of householders about iodized salt.

### 2.6. Data Processing and Analysis

The data were entered using EpiData version 3.1 and analyzed using SPSS version 20. Summary statistics like frequency, percent, mean, and standard deviation were used to describe data. Univariate and multivariate binary logistic regression analysis was used to assess the association between the outcome variable and independent variables. Multi-colinearity was checked for all independent variables using the variation inflation factor (VIF). Variables with *p* value=0.05 in the univariate analysis were entered into a multivariate binary logistic regression mode. Hosmer–Lemeshow goodness of fit was used to check the fitness of the final model. Adjusted odds ratio with 95% confidence interval was used to report the strength of associations. *p* value < 0.05 in the multivariate binary logistic analysis was used to declare statistical significance.

### 2.7. Ethical Considerations

Ethical clearance was obtained from the Ethical Review Committee of Madda Walabu University, Goba Referral Hospital, with the letter reference number 01/2/10256. Formal cooperation letter was written from the Madda Walabu University Goba Referral Hospital Academic and Research directorate to Kore woreda administration. After receiving permission from the respective woreda and town authorities, verbal informed consent was obtained from each head of the households before the interview and salt testing process. Health information about the use of iodized salt and handling practices was given to each respondent after data collection.

### 2.8. Operational Definitions

*Adequately Iodized Salt*. If the householders' salt samples had ≥15 ppm of iodized salt after testing with the rapid test kit.

*Knowledge about Iodized Salt*. A series of ten questions related to uses of iodized salt, sources of iodized salt, and knowledge of iodine deficiency-related disorders were asked. Score of “1” was given for correct answers and “0” for incorrect answers. Study participants who scored at least 50% on the knowledge questions were categorized as having good knowledge, and others were categorized as having poor knowledge.

## 3. Results

### 3.1. Sociodemographic Characteristics of Respondents

A total of 394 households were included in this study with a response rate of 100%. The mean age of respondents was 27 years with a standard deviation of 8.7 years. All of the household members who participated in food preparation were females (Table 1).

### 3.2. Knowledge of Respondents about Iodized Salt and Its Proper Handling

Out of all households, 214 (54.3%) of the households use unpacked dietary salt. Regarding the type of the container, 200 (50.8%) of the participants use a closed plastic container and 341 (86.5%) store dietary salt at a dry place. Concerning sunlight exposure, 358 (91.1%) of the study respondents did not expose their dietary salt to the sunlight. Out of all respondents, 174 (44.2%) add dietary salt to the food from the beginning of the cooking process (Table 2).

### 3.3. The Magnitude of Households with Adequately Iodized Salt

Out of all households, 223 (56.6%) had adequately iodized salt and the remaining 171 (43.4%) had inadequately iodized salt.

### 3.4. Factors Associated with the Availability of Adequately Iodized Dietary Salt

Multiple binary logistic regression analysis found that not exposing iodized salt to sunlight (AOR = 2.35, 95% CI: 1.1, 5.2), storing dietary salt at a dry and cold place [(AOR = 4.77, 95% CI:1.39, 16.45) and (AOR = 8.23, 95% CI: 1.44, 47.19), and having good knowledge about iodized salt (AOR = 1.88, 95% CI: 1.18, 3.01) were significantly associated with the presence of adequately iodized salt at household level (Table 3).

## 4. Discussion

This study aimed to assess the magnitude of households with adequately iodized dietary salt in the town of Kore, Southeast Ethiopia. The overall proportion of households using adequately iodized salt (≥15 parts per million) was found to be 56.6%. This finding is far below the World Health Organization target (≥90%) to eliminate iodine deficiency disorders [1]. The finding of this study reveals that the proportion of households with adequately iodized salt is lower than the study reports from South Wollo (68.8%) [21], Asella town (62.9%) [22], and Sidama Zone, Ethiopia (65%) [23]. In contrast, the finding of this study was higher compared to the reported result from Dabat district, North-west Ethiopia (33.2%) [24], Jijiga town, Eastern Ethiopia (26.6%) [25], Maychew, North Ethiopia (33%) [26], Lalo Asabi district, West Ethiopia (8.7%) [27], Gondar town (28.9%) [28], and Shebe-Senbo district, Ethiopia (26.2%) [29].

When we compare the coverage of adequately iodized salt in this study with coverage of other countries with similar socioeconomic setup, the current finding is lower than the coverage in India (60%) [30], Ghana (75.6%) [31], and South Africa (63%) [32]. But, it is slightly higher than the reports from Sudan (14.4%) [33] and Pakistan (15%) [34]. This variation could be related to a difference in the time the studies were conducted. As evidence from the Ethiopian demographic and health survey, the coverage of iodized salt increased from 28.4% [12] in 2000 to 89% in 2016 [16]. Even if the national surveys show that coverage of iodized salt at the household level is increased over the years, adequacy of the iodine level in the salt is still very low [15]. Additionally, it might be due to differences in availability and accessibility of iodized salt in the market, policies to fortify salt with iodine, and monitoring regarding utilization of iodized salt in those countries.

This study found that householders who did not expose dietary salt to the sunlight were more likely to have adequately iodized salt (AOR = 2.35 95% CI: 1.1–5.2) compared to their counterparts. This finding is supported by previous studies [21, 28, 35]. This might be due to the halogen nature of iodine, and its exposure to excess oxygen and carbon dioxide slowly oxidizes it to metal carbonate and elemental iodine which then evaporates [36].

This study also revealed that householders who store iodized salt at a dry or cold place were more likely to have adequately iodized salt compared to those who stored the iodized salt at moist places. This might be due to the volatility nature of iodine, and it needs optimum temperature and humidity to keep the iodine in the dietary salt stable [37].

A higher proportion of adequately iodized salt was observed among householders who had good knowledge about iodized salt and iodine deficiency disorders (AOR = 1.88 95% CI: 1.18–3.01) compared to their counterparts. This finding is in line with the study done in Dabat district, North-west Ethiopia [24], Asella town, east Arsi zone [22], and Sidama zone, South Ethiopia [23]. It is plausible that individuals with better knowledge tend to demand and consume iodized salt than those without adequate knowledge [31, 38].

This study assessed the magnitude and factors associated with availability of adequately iodized salt at the household level. The findings of this study should be used in light of the following limitations: the study used the rapid test kit to determine availability of iodine in the salt, and other quantitative methods like titration were not used. Moreover, the study used a cross-sectional study design, so it does not guarantee a cause-effect relationship between factors and outcome variables. Furthermore, seasonal change in the availability and accessibility of adequately iodized salt might not be addressed by this study due to the nature of the study. Finally, this study will not warrant the intake of sufficient iodine since we did not assess the urinary iodine concentration of the sampled population.

## 5. Conclusion

The availability of adequately iodized salt in the study area was far below the World Health Organization recommendation. Not exposing dietary salt to sunlight, storing iodized salt at a dry and cold place, and having good knowledge about iodized salt were significantly associated with the presence of adequately iodized salt at the household level. Information regarding the importance of iodized salt and proper handling of dietary salt should be communicated to the householders.

## Figures and Tables

**Table 1:** Sociodemographic characteristics of respondents in Kore Town, Southeast Ethiopia, 2019.

Variables	Availability of adequately iodized salt in the household		Cramer's V	Chi^2^*p* value
	Adequate, *n* (%)	Inadequate, *n* (%)		
*Age*			0.1	0.001
15–24 years	85 (48.0)	92 (52.0)		
25–34 years	86 (58.9)	60 (41.1))		
35–44 years	40 (78.4)	11 (21.6)		
≥45 years	12 (60.0)	8 (40.0)		

*Marital status*			0.03	0.8
Married	155 (57.6)	114 (42.4)		
Single	63 (54.8)	52 (45.2)		
Divorced/widowed	5 (50.0)	5 (50.0)		

*Ethnicity*			0.1	0.03
Oromo	185 (54.4)	155 (45.6)		
Amhara	29 (76.3)	9 (23.7)		
Others^a^	9 (56.2)	7 (43.8)		

*Religion*			0.2	<0.001
Muslim	157 (51.0)	151 (49.0)		
Orthodox	42 (76.4)	13 (23.6)		
Protestant	24 (77.4)	7 (22.6)		

*Family size of households*			0.07	0.4
2–6 persons	157 (57.5)	116 (42.5)		
7–11 persons	62 (56.4)	48 (43.6)		
12–16 persons	4 (36.4)	7 (63.4)		

*Average monthly income of the household head in ETB* ^b^			0.2	0.006
<1001	108 (50.5)	106 (49.5)		
1001–2000	40 (52.6)	36 (47.4)		
2001–3000	34 (69.4)	15 (30.6)		
3001–4000	18 (72.0)	7 (28.0)		
≥4001	23 (76.7)	7 (23.3)		

*Notes*. ^a^Wolaita, Hadiya, and Sidama; ^b^Ethiopian birr.

**Table 2:** Dietary salt handling practice of householders in Kore Town, Southeast Ethiopia, 2019.

Variables		Availability of adequately iodized salt in the household		Cramer's V	Chi^2^*p* value
		Adequate, *n* (%)	Inadequate, *n* (%)		
*Type of dietary salt*				0.2	<0.001
Unpacked^a^		100 (46.7)	114 (53.3)		
Packed		123 (68.3)	57 (31.7)		

*Type of container*				0.2	0.003
Plastic package		68 (70.1)	29 (29.9)		
Closed plastic cup		110 (55.0)	90 (45.0)		
Open plastic cup		45 (46.4)	52 (53.6)		

*Storage place*				0.2	0.01
Dry place		199 (58.4)	142 (41.6)		
Cold place		9 (69.2)	4 (30.8)		
Near cooking fire		11 (52.4)	10 (47.6)		
Moist place		4 (21.1)	15 (78.9)		

*Exposing salt to sunlight*				0.1	0.02
Yes		14 (38.9)	22 (61.1)		
No		209 (58.4)	149 (41.6)		

*Washing iodized salt*				0.1	0.1
Yes		16 (45.7)	19 (54.3)		
No		207 (57.7)	152 (42.3)		

*Types of the market to buy dietary salt*				0.2	<0.001
Open market		131 (50.6)	128 (49.4)		
Supermarket		95 (70.4)	40 (29.6)		

*Duration of storing dietary salt*				0.1	0.07
<2 months		214 (56.3)	166 (43.7)		
≥2 months		9 (64.3)	5 (35.7)		

*Time to add dietary salt to the cooked food*				0.3	<0.001
From the beginning of the cooking process		82 (47.1)	92 (52.9)		
In the middle of the cooking process		67 (51.1)	64 (48.9)	
At the end of the cooking process		74 (83.1)	15 (16.9)	

*Notes*. ^a^The salt is bought from the market without any package.

**Table 3:** Factors associated with the availability of adequately iodized dietary salt at household level in Kore Town, Southeast Ethiopia, 2019.

Variables	Availability of adequately iodized salt in the household		AOR^b^ (95% CI)
	Adequate, *n* (%)	Inadequate, *n* (%)	
*Knowledge about iodized salt*			
Poor knowledge	114 (53)	102 (47)	1
Good knowledge	57 (32)	121 (68)	1.88 (1.18, 3.01)^*∗*^

*Type of iodized salt*			
Unpacked^a^	114 (53.3)	100 (46.7)	1
Packed	57 (31.7)	123 (68.3)	1.33 (0.78, 2.27)

*Type of container for iodized salt*			
Bag in which salt is bought	29 (29.9)	68 (70.1)	1.16 (0.53, 2.55)
Closed plastic	90 (45)	110 (55)	1.07 (0.6, 1.9)
Opened plastic	52 (53.6)	45 (46.4)	1

*Exposure of iodized salt to sunlight*			
Yes	22 (61.1)	14 (38.9)	1
No	149 (41.6)	209 (58.4)	2.35 (1.1, 5.2)^*∗*^

*Storage place of iodized salt*			
Dry place	142 (41.6)	199 (58.4)	4.77 (1.39, 16.45)^*∗*^
Cold place	4 (30.8)	9 (69.2)	8.23 (1.44, 47.19)^*∗*^
Next to fire	10 (47.6)	11 (52.4)	2.95 (0.65, 13.45)
Moist place	15 (78.2)	4 (21.1)	1

*Notes*. ^*∗*^Statistically significant at *p* < 0.05. ppm: parts per million. ^a^The salt is bought from the market without any package. ^b^Adjusted for storage place, types of container, type of iodized salt, exposure to sunlight, income of householders, occupation of head of household, religion of head of household, and knowledge of household head about iodized salt.

## Data Availability

The data that support the findings of this study are available from the corresponding author upon reasonable request.
